# Supplementary material for: Effects of Quercitrin on PRV-Induced Secretion of Reactive Oxygen Species and Prediction of lncRNA Regulatory Targets in 3D4/2 Cells
Source: Antioxidants (Basel). 2022 Mar 25;11(4):631. doi: 10.3390/antiox11040631 (PMC9031018; doi:10.3390/antiox11040631)
Supplement: Supplementary file 1 [file antioxidants-11-00631-s001.zip › antioxidants-1614568-supplementary.pdf]

Supplymentary Table S1 Output statistics of the sequencing reads for each sample.

Sample	Raw reads	Clean reads	Clean bases(G)	Error rate(%)	Q20(%)	Q30(%)	GC(%)
**C1**	78545122	78347416	10.9	0.2	97.52	93.05	45.52
**C2**	75705446	75532868	10.51	0.19	97.73	93.58	45.56
**C3**	81726938	81504264	11.34	0.24	97.35	92.77	45.42
**P1**	76628834	76379484	10.62	0.28	97.24	92.57	46.36
**P2**	81128982	80933782	11.24	0.22	97.8	93.80	46.53
**P3**	75737372	75535648	10.51	0.23	97.62	93.34	46.59
**P+Q1**	81143544	80901170	11.27	0.26	97.43	92.98	46.24
**P+Q2**	85956418	85723402	11.93	0.24	97.62	93.37	45.99
**P+Q3**	98378380	98107478	13.66	0.25	97.51	93.13	46.00

Supplymentary Table S2 The 14 mRNA differentially expressed among the three groups

Genbank#	**Accession number**	**Symbol**
**ENSSSCG00000004751**	100621371	CHP1
**ENSSSCG00000006580**	HGNC:10492	S100A2
**ENSSSCG00000009560**	100621036	TFDP1
**ENSSSCG00000011264**	100519275	CSRNP1
**ENSSSCG00000013433**	100512586	ADAMTSL5
**ENSSSCG00000013457**	100738665	DOT1L
**ENSSSCG00000014335**	100520367	REEP2
**ENSSSCG00000016438**	100517250	NUB1
**ENSSSCG00000016991**	100522469	DUSP1
**ENSSSCG00000021569**	100626072	MMP25
**ENSSSCG00000030209**	102161832	MFNG
**ENSSSCG00000031262**	733688	TXNIP
**ENSSSCG00000031342**	110261334	-
**ENSSSCG00000033126**	110257088	FAM168B
